# CD137 Costimulation Enhances the Antitumor Activity of Vγ9Vδ2-T Cells in IL-10-Mediated Immunosuppressive Tumor Microenvironment

**DOI:** 10.3389/fimmu.2022.872122

**Published:** 2022-06-17

**Authors:** Yujun Pei, Zheng Xiang, Kun Wen, Chloe Ran Tu, Xiwei Wang, Yanmei Zhang, Xiaofeng Mu, Yinping Liu, Wenwei Tu

**Affiliations:** ^1^Department of Paediatrics and Adolescent Medicine, Li Ka Shing Faculty of Medicine, The University of Hong Kong, Hong Kong, Hong Kong SAR, China; ^2^Bioland Laboratory (Guangzhou Regenerative Medicine and Health Guangdong Laboratory), Guangzhou, China; ^3^Computational and Systems Biology Interdepartmental Program, University of California, Los Angeles, Los Angeles, CA, United States

**Keywords:** CD137, γδ-T cells, antitumor acitivity, IL-10, immunotherapy

## Abstract

Although γδ-T cell-based tumor immunotherapy using phosphoantigens to boost γδ-T cell immunity has shown success in some cancer patients, the clinical application is limited due to the rapid exhaustion of Vγ9Vδ2-T cells caused by repetitive stimulation from phosphoantigens and the profoundly immunosuppressive tumor microenvironment (TME). In this study, using a cell culture medium containing human and viral interleukin-10 (hIL-10 and vIL-10) secreted from EBV-transformed lymphoblastoid B cell lines (EBV-LCL) to mimic the immunosuppressive TEM, we found that the antitumor activity of Vγ9Vδ2-T cells was highly suppressed by endogenous hIL-10 and vIL-10 within the TME. CD137 costimulation could provide an anti-exhaustion signal to mitigate the suppressive effects of IL-10 in TME by suppressing IL-10R1 expression on Vγ9Vδ2-T cells. CD137 costimulation also improved the compromised antitumor activity of Vγ9Vδ2-T cells in TME with high levels of IL-10 in Rag2^-/-^ γc^-/-^ mice. In humanized mice, CD137 costimulation boosted the therapeutic effects of aminobisphosphonate pamidronate against EBV-induced lymphoma. Our study offers a novel approach to overcoming the obstacle of the hIL-10 and vIL-10-mediated immunosuppressive microenvironment by costimulating CD137 and enhancing the efficacy of γδ-T cell-based tumor therapy.

## Introduction

Epstein-Barr virus (EBV) is a predominant type of human herpesviruses. It infects over 95% of the population by adulthood (1, 2). EBV infection is highly correlated with several human malignancies (1–3). As the first known human tumor virus, the carcinogenesis of EBV has been identified in various hematopoietic and epithelial cell cancers, including EBV-associated tumors and lymphoproliferative disorder (1, 2, 4). Current therapeutic approaches for EBV-associated tumors are restricted by undesirable side effects and ineffectiveness for refractory or relapsed diseases (5, 6). It was reported that EBV-specific CTL-based therapy is effective in the control of EBV-associated malignancy (5, 6). However, its clinical application is hampered due to insufficient quantity of EBV-specific CTL generated *ex vivo* (7).

As a major subset of human γδ-T cells, Vγ9Vδ2-T cells have been extensively demonstrated to have promising anti-tumor effects (8–13). Vγ9Vδ2-T cells can be activated specifically by phosphoantigens from isoprenoid biosynthesis in an MHC-unrestricted manner. Aminobisphosphonates pamidronate (PAM) and zoledronate (ZOL) are commonly used pharmacological phosphoantigens for osteoporosis and Paget’s disease treatment (11, 14). Previously, we demonstrated that direct administration of PAM could expand Vγ9Vδ2-T cells *in vivo* and thus control EBV-induced lymphoma in humanized mice, suggesting that Vγ9Vδ2-T cell-based immunotherapy is promising for treating EBV-associated tumors (15). A recent meta-analysis of about 18,000 human cancers revealed that tumor-infiltrating γδ T cells are the most favorable cancer-wide prognostic marker (16). However, the clinical application was limited by the rapid exhaustion of Vγ9Vδ2-T cells caused by the repetitive stimulation from phosphoantigens *in vivo* (17) and the profoundly immunosuppressive tumor microenvironment (TME) (18–20).

Interleukin (IL)-10, as a major immunosuppressive cytokine in TME secreted by tumor cells, can help tumor cells escape immunological recognition and destruction (21–24). Current evidence indicates that EBV codes a homologue of human IL-10 (vIL-10) with immunosuppressive properties to evade immunity and establish persistent/latent infections (25–28). EBV-LCL also express and release various amounts of human IL-10 (hIL-10) (29, 30). hIL-10 and vIL-10 are crucial for B cell transformation of B cell (31, 32) and oncogenesis of EBV-associated tumors (33). However, whether the antitumor activity of Vγ9Vδ2-T cells was suppressed by IL-10 in TME remained largely unknown.

CD137 (4-1BB), a membrane-bound receptor, is a costimulatory molecule expressed in many lymphocytes (34–36). Recently, we demonstrated that CD137 costimulation enhanced the activation and cytolytic activity of Vγ9Vδ2-T cells against virus-infected cells (37). Importantly, boosting cancer immunotherapy with agonistic CD137 antibodies has been demonstrated to be a promising therapeutic strategy for different tumors (38, 39). However, the roles of CD137 signaling for human Vγ9Vδ2-T cells in the immunosuppressive TME remained to be determined.

In this study, we aim to clarify whether IL-10 in the TME is responsible for the exhaustion of Vγ9Vδ2-T cells and determine whether targeting CD137 can enhance the antitumor activity of Vγ9Vδ2-T cells compromised by the immunosuppressive TME.

## Materials and Methods

### Vγ9Vδ2-T Cell Cultures

hPBMC were isolated from buffy coats by Ficoll-Hypaque gradient centrifugation of EBV^+^ healthy donors after informed consents were obtained. PAM-expanded Vγ9Vδ2-T cells were prepared according to the protocol we established before (40). Briefly, hPBMC were cultured in RPMI1640 medium with 10% fetal bovine serum (FBS) in the presence of PAM from day 0 to day 3 at a concentration of 9μg/ml. Recombinant human IL-2 was added to medium from day 3 to day 14 at a concentration of 500 IU/ml. After 2 weeks, the γδ-T cells were purified by positive selection with α-TCRγ/δ MicroBead (Miltenyi Biotec).

### Cytotoxic Assay

Purified Vγ9Vδ2-T cells were cultured with IL-10^low^ or IL-10^high^ conditioned medium for 24h, RPMI 1640 with 10% FBS medium (plain medium, PM) as a control. The pretreated Vγ9Vδ2-T cells (effector cells, E) were cocultured with autologous EBV-LCL (target cells, T) at an E: T ratio of 10:1 for 4 to 6 h in the IL-10^low/high^ CM or PM, and then the death of target cells was analyzed with flow cytometry. Cells were stained with anti-CD3 to identify Vγ9Vδ2-T cells and propidium iodide (PI) was used to identify dead cells. The death of EBV-LCL was shown as the percentage of PI^+^ cells in the CD3^-^ population (40). In some experiments, neutralizing antibody against IL-10 (abcam) was added to block IL-10 mediated pathways. To confirm the suppressive role of IL-10 in the CM, recombinant hIL-10 (Peprotech) or recombinant vIL-10 (R&D systems) was added to culture medium at the indicated concentration.

### Establishment of EBV-LCL

EBV-secreting cell lines B95-8 and B95.8EBfaV-GFP were cultured and EBV-containing supernatants were collected for the following infection. hPBMC were incubated with EBV-containing supernatants, and then cultured in the RPMI 1640 medium containing 15% FBS with the addition of cyclosporine-A (1μg/ml) as we describe before (15).

### Collection of EBV-LCL Conditioned Medium

EBV-LCL were cultured in RPMI1640 medium for 24 h. The conditioned medium (CM) was collected, centrifuged at 5000 rpm at 4°C for 10 min to remove cell debris and then frozen at −80°C in aliquots. Stored CM was passed through a 0.22-μm syringe filter (Millipore) before use. Plain medium (PM) collected from complete medium without cell incubation under the same experimental conditions served as the control for CM.

### Determination of hIL-10 and vIL-10 Levels

For hIL-10, the concentrations in conditioned medium were measured by ELISA. The procedures for human IL-10 ELISA kits (Biolegend, San Diego, CA, USA) were performed based on the manufacturer’s instructions. For vIL-10, the concentrations in conditioned medium were measured according to the method described before (41). The conditioned medium was concentrated by Amicon-Ultra centrifugation filters (Millipore) following the manufacturer’s instructions. Then, the concentrated conditioned medium was used for performing Western blot assay. Mouse monoclonal antibody against vIL-10 (R&D) was used as primary antibody for incubating transferred membranes at 4°C overnight. Horseradish peroxide conjugated goat anti-mouse secondary antibody (R&D) was used as secondary antibody for detecting vIL-10 levels. The bands of Western blot were quantified by “Gels” analysis tool of ImageJ. Recombinant vIL-10 was used as a standard to quantify the vIL-10 level in conditioned medium.

### Establishment and Treatment of EBV-Associated Lymphoma in Mice

All animal studies were approved and performed in compliance with the guidelines for the use of experimental animals by the Committee on the Use of Live Animals in the Teaching and Research, the University of Hong Kong. Rag2^−/−^γc^−/−^ mice were bred in Centre for Comparative Medicine Research of the University of Hong Kong. Humanized mice were generated according to the protocol we established before (14, 15). Rag2^−/−^γc^−/−^ or humanized mice were inoculated with EBV-LCL expressing high or low level of IL-10 (0.1×10^6^/mouse) by subcutaneous injection to establish the EBV-associated lymphoma model. For Rag2^−/−^γc^−/−^ mice, PAM-expanded Vγ9Vδ2-T cells (5×10^6^/mouse) with or without the addition of SA-hCD137L (5μg/mouse) were adoptively transferred intravenously into EBV-associated lymphoma murine model at indicated time. For humanized mice, PAM (5mg/kg body weight) and SA-hCD137L (15μg/mouse) were injected intraperitoneally at the indicated time. The mice treated with an equivalent volume of PBS or SA were used as controls. The tumor volume and mice survival were monitored every day and calculated at the indicated time. Mice were counted as dying when their subcutaneous tumor diameter was larger than 17 mm and thus sacrificed according to the regulation of Centre for Comparative Medicine Research of the University of Hong Kong. Otherwise, mice were monitored for 100 days before being sacrificed. The tumor tissues were reserved for immunohistochemical evaluation.

### Preparation of the Recombinant SA-hCD137L Protein

Recombinant SA-hCD137L proteins were generated as described before (37). Briefly, the DNA sequences were synthesized encoding the extracellular domain of human CD137L (a.a. 58-254) and the core streptavidin (SA; a.a. 16-133) with an N-terminal 6×His tag. The recombinant SA-hCD137L protein was expressed in *E. coli* by inserting the SA-hCD137L DNA fragments into the pETH expression vector and transforming into competent cells. After purifying with Ni-nitrilotriacetic acid affinity chromatography (QIAGEN, Germany), the recombinant SA-hCD137L protein was filtered a and quantitated by BCA Protein Assay Kit (Pierce, USA).

### Flow Cytometric Analysis

Cells were stained for surface molecules with the following antibodies: αIL10R (Miltenyi Biotec, clone REA239), αCD3 (Biolegend, clone HIT3a), αTCRγ9 (Biolegend, clone B3), αTCRVδ2 (Biolegend, clone B6), and αCD137 (Biolegend, clone 4B4-1). All samples were performed with a FACS LSR II (BD). The results were analyzed with FlowJo software.

### Histological Staining and Immunohistochemical Assays

The tumor tissues were fixed with 10% formalin for 24 h and maintained in 70% ethanol. Fixed tumor tissues were embedded in paraffin and sectioned. The tumor sections were performed immunohistochemistry staining with αIL-10 antibody (abcam) (42).

### Statistics

Data are shown in the form of mean ± standard error of the mean (SEM). All data were tested by Shapiro-Wilk test to verify the normality. For data that did not meet normal distribution, Mann-Whitney U test was used for analysis. For data that met normal distribution, one-way analysis of variance (ANOVA) with Bonferroni correction was used for analysis. For multiple variables, two-way ANOVA was used. Kaplan-Meier log-rank test was used for comparing survival among different groups. Two-tailed test was used for all analyses. P < 0.05 was regarded as significant.

## Results

### Antitumor Activity of Vγ9Vδ2-T Cells Was Inhibited by IL-10 Secreted From EBV-LCL *In Vitro*

**Figure 1:**
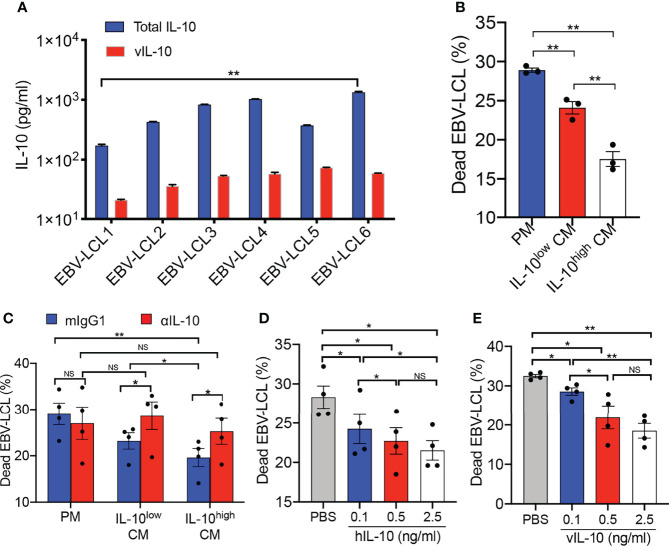
Antitumor activity of Vγ9Vδ2-T cells was inhibited by IL-10 secreted from EBV-LCL *in vitro*. **(A)** The concentration of vIL-10 and total IL-10 in conditioned medium (CM) collected from EBV-LCL established from different donors were detected. **(B)** Purified Vγ9Vδ2-T cells were pretreated in the IL-10^low^ CM and IL-10^high^ CM separately for 24 h, RPMI 1640 with 10% FBS medium (plain medium, PM) as a control. Pretreated Vγ9Vδ2-T cells then cocultured with autologous EBV-LCL at an effector: target (E:T) ratio of 10:1 for 4–6 h in the IL-10^low/high^ CM and PM, respectively. Cytotoxicity was calculated as the proportion of dead EBV-LCL (CD3^-^PI^+^). **(C)** Purified Vγ9Vδ2-T cells were pretreated with IL-10^low^ CM, IL-10^high^ CM or PM in the presence of a neutralizing anti-IL-10 mAb (αIL-10, 5μg/ml) or isotype control (mIgG1, 5μg/ml), then cocultured with autologous EBV-LCL at an E:T ratio of 10:1 for 4–6 h in the IL-10^low/high^ CM and PM respectively. Cytotoxicity was calculated as the proportion of dead EBV-LCL (CD3^-^ PI^+^). **(D, E)** Purified Vγ9Vδ2-T cells were pretreated with recombinant hIL-10 **(D)** or vIL-10 **(E)** at different concentration, then cocultured with autologous EBV-LCL at an E:T ratio of 10:1 for 4–6h. The proportion of dead EBV-LCL (CD3^-^ PI^+^) were detected by flow cytometry. All data are shown as mean ± SEM and representative of three independent experiments. *p < 0.05; **p < 0.01; ns, no significant difference.

To investigate the effects of IL-10 in TME on the antitumor activity of Vγ9Vδ2-T cells, conditioned medium (CM) was obtained by collecting the supernatant of EBV-LCL culture for modeling TME *in vitro*. As shown in **Figure 1A**, CM from EBV-LCL culture established from different donors contained distinct levels of IL-10, and vIL-10 accounted for about 9.56 ± 5.74% of total IL-10. CM collected from EBV-LCL1 and EBV-LCL6, which contained the lowest and highest concentrations of IL-10, was used as IL-10^low^ CM and IL-10^high^ CM, respectively, in the following experiments. Importantly, the cytotoxic activity of IL-10^high^ CM-treated Vγ9Vδ2-T cells against EBV-LCL was significantly lower than IL-10^low^ CM- or PM-treated Vγ9Vδ2-T cells (**Figure 1B**). To verify the immunosuppressive role of IL-10 in the CM, an IL-10 neutralizing mAb was applied to block IL-10 signaling during Vγ9Vδ2-T cells exposed to IL-10^high/low^ CM. The reduced cytotoxicity of Vγ9Vδ2-T cells against EBV-LCL was significantly abrogated when blocked with the IL-10 neutralizing mAb (**Figure 1C**). Furthermore, both hIL-10 and vIL-10 recombinant proteins showed dose-dependent inhibitions in the cytotoxicity of Vγ9Vδ2-T cells against EBV-LCL in the PM (**Figures 1D, E**). Taken together, our data indicate that the antitumor activity of Vγ9Vδ2-T cells against EBV-LCL was suppressed by both the hIL-10 and vIL-10 in the CM from EBV-LCL *in vitro*.

### Antitumor Activity of Vγ9Vδ2-T Cells Against EBV-Induced Lymphoma Was Decreased Under IL-10^high^ TME *In Vivo*

**Figure 2:**
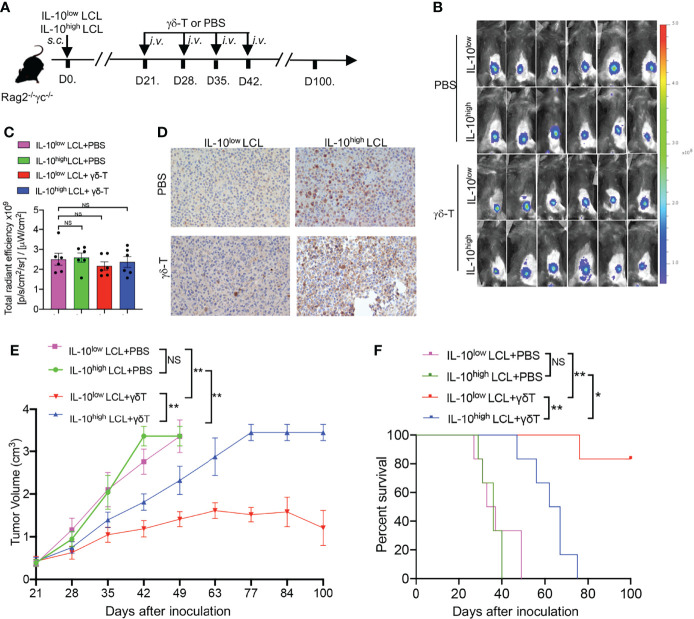
Antitumor activity of Vγ9Vδ2-T cells against EBV-induced lymphoma was decreased under IL-10^high^ TME *in vivo*. **(A)** IL-10^low^ LCL and IL-10^high^ LCL were injected *s.c.* in Rag2^−/−^γc^−/−^ mice separately. After 21 days, mice that had developed subcutaneous tumor were randomly divided into two groups respectively followed by the treatment with allogeneic Vγ9Vδ2-T cells or PBS at indicated time (six mice per group). **(B, C)** Whole-body fluorescence images **(B)** and total radiant efficiency **(C)** of mice before treatment with Vγ9Vδ2-T cells or PBS. **(D)** Representative histology of IL-10 in tumor sections that developed from IL-10^low^ LCL and IL-10^high^ LCL. **(E, F)** The tumor volume **(E)** and mouse survival **(F)** were determined at the indicated time. The tumor volume was compared using two-way ANOVA analysis, and mice survival was compared using Kaplan-Meier log-rank test. Data are representative for three independent experiments. *p < 0.05; **p < 0.01; ns, no significant difference.

To determine whether the therapeutic effects of Vγ9Vδ2-T cells on EBV-induced B cell lymphoma were inhibited by IL-10 within the TME, EBV-LCL1 expressing low levels of IL-10 (IL-10^low^ LCL) and EBV-LCL6 expressing high levels of IL-10 (IL-10^high^ LCL) were inoculated into Rag2^-/-^ γc^-/-^ mice, respectively (**Figure 2A**). After 21 days, large subcutaneous tumors developed in all the mice as detected by *in vivo* imaging (**Figures 2B, C**). The expressions of IL-10 in the tumor tissues generated from IL-10^low^ LCL and IL-10^high^ LCL were detected by immunohistochemistry (**Figure 2D**). Consistent with our previous results (15), Vγ9Vδ2-T cell treatment constrained tumor growth and prolonged the survival of tumor-bearing mice in contrast with the mice treated with PBS as the control (**Figures 2E, F**). Importantly, Vγ9Vδ2-T cells showed less efficacy in controlling EBV-induced lymphoma developed from IL-10^high^ LCL compared with that developed from IL-10^low^ LCL, along with larger tumor volume and lower survival rates (**Figures 2E, F**). These results suggest that the decreased antitumor activity of Vγ9Vδ2-T cells against EBV-induced lymphoma may be associated with IL-10^high^ TME *in vivo*.

### CD137 Costimulation Suppressed IL-10R1 Expression and Restored the Antitumor Activity of Vγ9Vδ2-T Cells

**Figure 3:**
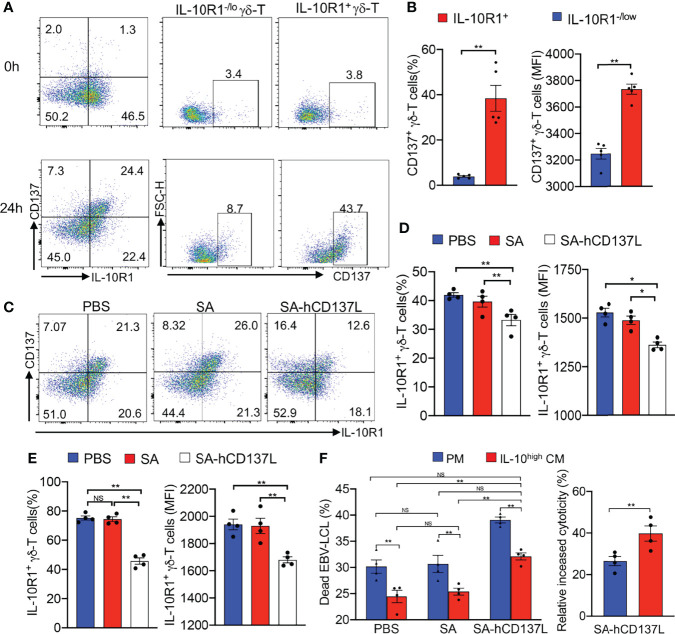
CD137 costimulation suppressed IL-10R1 expression and restored the antitumor activity of Vγ9Vδ2-T cells. **(A)** The expression of CD137 on IL-10R1^-/lo^ and IL-10R1^+^ Vγ9Vδ2-T cells before (0h) and after stimulation with anti-γδ-TCR mAb for 24h (24h) in IL-10^high^ CM. **(B)** The percentages and expression levels (mean fluorescence intensities, MFI) of CD137 on IL-10R1^-/lo^ and IL-10R1^+^ Vγ9Vδ2-T cells upon stimulation for 24h in IL-10^high^ CM. **(C, E)** The FACS patterns of CD137 and IL-10R1 expressions **(C)**, surface expression of IL-10R1 in total Vγ9Vδ2-T cells **(D)** and in CD137^+^ Vγ9Vδ2-T cells **(E)** upon stimulation by anti-γδ-TCR mAb supplemented with SA-CD137L (500ng/ml), PBS and SA in IL-10^high^ CM for 24h were detected by flow cytometry after surface staining of IL-10R1. **(F)** Purified Vγ9Vδ2-T cells were pretreated with SA-hCD137L (500ng/ml), PBS or SA for 24 h in the PM and IL-10^high^ CM, and then cocultured with autologous EBV-LCL at an E: T ratio of 10:1 for 4-6h. The proportions of dead EBV-LCL (CD3^-^ PI^+^, left), and the relative increase of cytotoxicity after being treated with SA-hCD137L (right) are shown. All data are shown as mean ± SEM and representative of three independent experiments. *p < 0.05; **p < 0.01; ns, no significant difference.

IL-10 mediates its biological effects mainly through a heterodimeric membrane receptor composed of IL-10R1 and IL-10R2 (43). Since IL-10R2 is shared by more than five IL-10 family cytokines (44), we investigated the expression of IL-10R1 on Vγ9Vδ2-T cells exposed to the IL-10^high^ CM upon γδ-TCR activation *in vitro*. Importantly, we found that following activation, IL-10R1^+^ Vγ9Vδ2-T cell subset expressed high levels of CD137 compared with IL-10R1^-/lo^ Vγ9Vδ2-T cell subset in the IL-10^high^ CM, indicating that CD137 could be an effective costimulatory signaling to restore the antitumor activity of Vγ9Vδ2-T cells compromised by the IL-10 in TME (**Figures 3A, B**).

To determine whether CD137 costimulation could provide an anti-exhaustion signal to mitigate the inhibiting effects mediated by IL-10 in TME, a recombinant SA-hCD137L protein containing a core streptavidin (SA) molecule with the extracellular domains of human CD137L (hCD137L) was generated as we reported previously (37). We found that the addition of the recombinant SA-hCD137L protein significantly inhibited the surface expression of IL-10R1 in total and CD137^+^ Vγ9Vδ2-T cells in IL-10^high^ CM in terms of both percentage and expression level (MFI) changes (**Figures 3C–E**). These results indicate that CD137 costimulation suppressed IL-10R1 expression in CD137^+^ Vγ9Vδ2-T cells, and thereby was able to reduce their sensitivity to endogenous IL-10 in the immunosuppressive TME.

To determine whether CD137 costimulation could rescue the impaired antitumor efficacy of Vγ9Vδ2-T cells in suppressive TME, the recombinant SA-hCD137L protein was added to the coculture of Vγ9Vδ2-T cells with EBV-LCL in IL-10^high^ CM for mimicking the tumor milieu. As shown in **Figure 3F**, the SA-hCD137L protein significantly increased the cytotoxicity of Vγ9Vδ2-T cells against EBV-LCL under both the immunosuppressive and normal microenvironments mimicked by the IL-10^high^ CM and the PM. Importantly, CD137 costimulation not only completely restored the reduced cytotoxicity of Vγ9Vδ2-T cells in the IL-10^high^ CM to normal levels, but also had a better effect to enhance the cytotoxic activity of Vγ9Vδ2-T cells in IL-10^high^ CM than that in PM (**Figure 3F**). These data demonstrate that CD137 engagement enables Vγ9Vδ2-T cells to withstand the hostile environment mediated by endogenous IL-10, resulting in the increase of the antitumor activity of Vγ9Vδ2-T cells *in vitro*.

### CD137 Costimulation Enhanced the Compromised Antitumor Activity of Vγ9Vδ2-T Cells With IL-10^high^ TME in Rag2^-/-^ γc^-/-^ Mice

**Figure 4:**
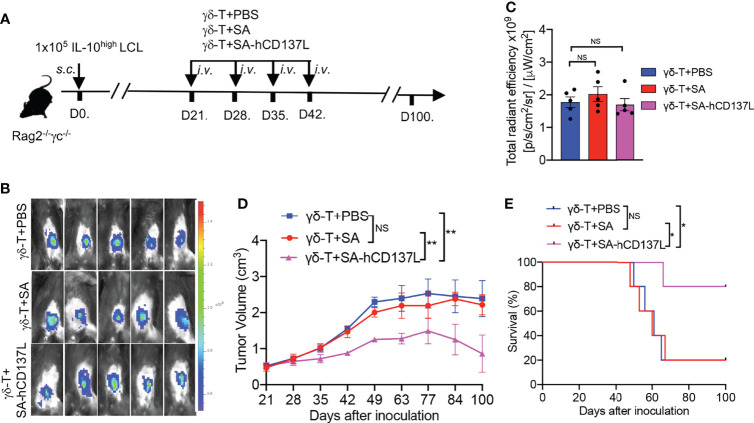
CD137 costimulation enhanced the compromised antitumor activity of Vγ9Vδ2-T cells with IL-10^high^ TME in Rag2^-/-^ γc^-/-^ mice. **(A)** Protocol for evaluation of the synergistic therapeutic effect of Vγ9Vδ2-T cells and SA-hCD137L on EBV-induced lymphoma in Rag2^-/-^ γc^-/-^ mice (five mice per group). **(B, C)** Whole-body fluorescence images **(B)** and total radiant efficiency **(C)** of mice before treatment with PAM, SA-hCD137, SA, and PBS. **(D, E)** The tumor volume **(D)** and mouse survival **(E)** were determined at the indicated time. The tumor volume was analyzed by two-way ANOVA test, and mice survival was analyzed by Kaplan-Meier log-rank test. Data are representative for three independent experiments. *p < 0.05; **p < 0.01; ns, no significant difference.

Previously we have demonstrated that Vγ9Vδ2-T cells could control EBV-inducing lymphoma (15), and their antitumor activity in controlling EBV-induced lymphoma developed from IL-10^high^ LCL was lower than that developed from IL-10^low^ LCL (**Figures 2E, F**). To further elucidate the roles of CD137 costimulation in the compromised antitumor activity of Vγ9Vδ2-T cells in IL-10^high^ TME *in vivo*, EGFP-expressing IL-10^high^ LCL was inoculated in Rag2^-/-^ γc^-/-^ mice, *s.c.* (**Figure 4A**). Twenty-one days later, mice bearing subcutaneous tumors were randomly divided into three groups as detected by *in vivo* imaging (**Figure 4B**). No significant differences were found in fluorescent density from tumor cells among the three groups after 21 days of tumor cell inoculation (**Figure 4C**). PAM-expanded Vγ9Vδ2-T cells were adoptively transferred to one group of the tumor-bearing mice with the recombinant SA-hCD137L protein weekly from day 21 to day 42. The other two groups of mice were adoptively transferred with Vγ9Vδ2-T cells in the presence of PBS or SA as the controls. Importantly, Vγ9Vδ2-T cells in combination with SA-hCD137L treatment significantly limited tumor growth (**Figure 4D**) and improved mouse survival (**Figure 4E**) compared to treatments of Vγ9Vδ2-T cells with PBS or SA. These data indicate that the costimulation of CD137 efficiently enhanced the antitumor activity of Vγ9Vδ2-T cells in the highly immunosuppressive microenvironment mediated by IL-10 *in vivo*.

### CD137 Costimulation Improved the Therapeutic Effect of PAM in Controlling EBV-Induced Lymphoma With IL-10^high^ TME in Humanized Mice

**Figure 5:**
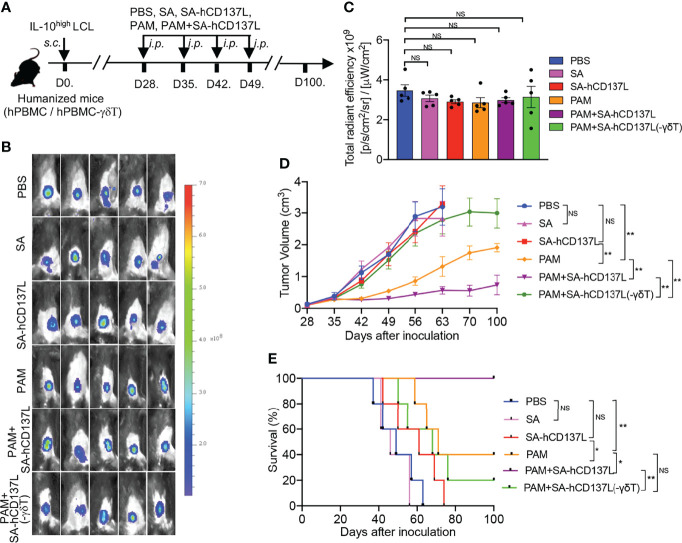
CD137 costimulation improved the therapeutic effect of PAM in controlling EBV-induced lymphoma with IL-10^high^ TME in humanized mice. **(A)** The evaluation protocol of the synergistic therapeutic effect of PAM and SA-hCD137L on EBV-induced lymphoma in humanized mice (five mice per group). **(B, C)** Whole-body fluorescence images **(B)** and total radiant efficiency **(C)** of mice before treatment with PAM, SA-hCD137, SA and PBS. **(D, E)** The tumor volume **(D)** and mouse survival **(E)** were determined at the indicated time. The tumor volume was analyzed by two-way ANOVA test, and mice survival was analyzed by Kaplan-Meier log-rank test. Data are representative for three independent experiments. *p < 0.05; **p < 0.01; ns, no significant difference.

Previously we had demonstrated that PAM could expand Vγ9Vδ2-T cells *in vivo* to control EBV-induced lymphoma in humanized mice with functional hPBMC (15). We then investigated the role of CD137 costimulation on the therapeutic effect of PAM in controlling EBV-induced lymphoma with IL-10^high^ TME in humanized mice. EBV-induced lymphoma with IL-10^high^ TME model was generated by inoculation *s.c.* of IL-10^high^ EBV-LCL in humanized mice (**Figure 5A**) (15). All humanized mice developed subcutaneous tumors after IL-10^high^ EBV-LCL inoculation for 28 days with similar fluorescent density from tumor cells as detected by *in vivo* imaging (**Figures 5B, C**). PAM, SA-hCD137L, or the combination of these two agents were injected intraperitoneally (*i.p.*) at days 28, 35, 42, and 49 after IL-10^high^ EBV-LCL inoculation (**Figure 5A**). PBS- and SA-treated mice were controls. As a result, PAM administration alone decreased the tumor volume significantly and extended the survival of the tumor-bearing humanized mice compared with the treatment with PBS, SA, or SA-hCD137L protein alone, respectively (**Figures 5D, E**). Importantly, the combination treatment of PAM with SA-hCD137L was more potent than PAM alone to control the development of EBV-induced lymphoma with IL-10^high^ TME in humanized mice, in terms of tumor growth and survival (**Figures 5D, E**).

Humanized mice reconstituted with Vγ9Vδ2-T-cell-depleted hPBMC were also used to confirm whether the effect of SA-hCD137L costimulation on the control of EBV-induced lymphoma with IL-10^high^ TME was mediated by Vγ9Vδ2-T cells (**Figure 5A**). As shown in **Figures 5D, E**, there were no therapeutic effects by the combination treatment of PAM with SA-hCD137L in humanized mice reconstituted with Vγ9Vδ2-T-cell-depleted hPBMC. These data demonstrate that the recombinant SA-hCD137L protein had a synergistic effect with PAM to overcome the barriers of IL-10^high^ TME *in vivo* and this synergistic effect was mainly mediated by Vγ9Vδ2-T cells.

## Discussion

In this study, we demonstrated that the antitumor activities of Vγ9Vδ2-T cells against EBV-LCL were inhibited by both hIL-10 and vIL-10 *in vitro* and *in vivo*. Importantly, we found that IL-10R1 was highly expressed on CD137^+^ Vγ9Vδ2-T cells compared to CD137^-/lo^ Vγ9Vδ2-T cells following activation. CD137 engagement significantly suppressed IL-10R1 expression in Vγ9Vδ2-T cells, therefore reducing the Vγ9Vδ2-T cells’ sensitivity to IL-10 in the TME. We further demonstrated that SA-hCD137L in a tetrameric form of human CD137L protein obviously enhanced the therapeutic effects of adoptive transfer of *ex vivo* expanded Vγ9Vδ2-T cell in Rag2^-/-^γc^-/-^ mice and direct administration of PAM in humanized mice for the treatment of EBV-induced lymphoma with IL-10^high^ TME.

IL-10 is important as an immunoregulatory cytokine to suppress inflammatory responses. However, its effects on tumorigenesis and development are controversial (45). IL-10 can inhibit the process of antigen presentation by downregulating the expression of MHC-II in APCs (46) and MHC-I in tumor cells (47). Thus, IL-10 can facilitate tumor escape by contributing to an immunosuppressive environment. A meta-analysis of 1788 cancer patients also revealed that the elevated serum IL-10 can predict poor prognosis (48). Paradoxically, it was reported that IL-10 can also induce immune-dependent antitumor effects (15, 49–51). Therefore, the roles of IL-10 on tumor development are dependent on the local environment and physiopathological states. The inhibitory role of IL-10 on APCs, CD8^+^ T cells, and CD4^+^ T cells has been clearly defined, but its impact on Vγ9Vδ2-T cells remains unclear. In this study, our data supported that hIL-10 and vIL-10 derived from tumor cells and EBV significantly inhibited the cytotoxicity of Vγ9Vδ2-T cells, which substantially limits the antitumor efficacy of Vγ9Vδ2-T cells.

EBV has evolved to express vIL-10, thereby providing a suitable microenvironment for itself to evade immunity and cause tumorigenesis (52, 53). The structurally homologous viral and human IL-10 perform similarly in several biological properties, including inhibition of IFN-γ production, suppression of T cell proliferation in response to antigens and mitogens, and stimulation of B cell growth (54). This similarity has raised the possibility that EBV might have captured the IL-10 gene during evolution. Furthermore, IL-10 has been shown to be involved in the pathogenesis of lymphoid disorders (55, 56). Elevated IL-10 levels are correlated with shorter survival and adverse disease features in patients with EBV-associated tumors (33, 57). Thus, we reasoned that hIL-10 and vIL-10 may be associated with the suppression of Vγ9Vδ2-T cells’ antitumor activity. Such an interaction would provide a suitable microenvironment for viruses to evade immunity and cause tumorigenesis. Here, our *in vitro* data revealed that vIL-10 derived from EBV and hIL-10 derived from EBV-LCL were the dominant factors for inhibiting the antitumor activity of Vγ9Vδ2-T cells in TME. Our *in vivo* data also suggested that the reduced antitumor activity of Vγ9Vδ2-T cells against EBV-induced lymphoma may be associated with IL-10^high^ TME. Further study using IL-10 neutralizing mAb or IL-10 knockout mice is required to determine the exact role of IL-10 in antitumor activity of Vγ9Vδ2-T cells *in vivo*. Of note, additional factors in the CM might also contribute to suppressing Vγ9Vδ2-T cells activity because a smaller extent of cytotoxicity reduction after treatment with recombinant hIL-10 and vIL-10 proteins was observed when compared with IL-10^high^ CM (**Figure 1**).

Recently, clinical trials utilizing bisphosphonates, such as PAM and ZOL, to expand γδ-T cell *in vivo* in combination with IL-2 therapy or adoptive transfer of *ex vivo* cultured γδ-T cells were performed in patients with tumors and virus infections (11, 15, 58, 59). Administration of bisphosphonates with IL-2 and the transfer of expanded autologous Vγ9Vδ2 T-cells have been demonstrated to be safe with limited adverse events (60). However, there is only a modest efficacy in the treatment of some tumors (61, 62). One drawback of γδ-T cell-based immunotherapy is the rapid exhaustion of proliferation and effector responses due to repeated phosphoantigen treatments (17). Another drawback of this therapy is the impaired antitumor activity of γδ-T cells caused by the tumor immunosuppressive microenvironment (18, 63).

CD137 is a promising costimulatory immunologic target for enhancing antitumor immune responses (39). CD137 costimulation, known as “stepping on the accelerator,” is believed to be a compelling complement for “removing the brakes” *via* blocking inhibitory signaling. Importantly, it is now appreciated that CD137 signaling not only works as an “accelerator” to provide costimulation, but also breaks and reverses the established anergy in cytotoxic T lymphocytes (CTLs) (64, 65). However, the role of CD137 signaling in Vγ9Vδ2-T cells within the context of IL-10-mediated TME is not clear. Here, we revealed that IL-10R1^+^ Vγ9Vδ2 T-cell subset expressed high levels of CD137. Moreover, CD137 costimulation suppressed IL-10R1 in Vγ9Vδ2-T cells, suggesting that CD137 engagement possessed the potential to ameliorate the exhaustion and dysfunction of Vγ9Vδ2-T cells.

Ligation of CD137 is correlated with effective antitumor responses; however, the application of anti-CD137 agonistic antibodies in patients is limited by a variety of side effects (66). The natural CD137 ligand is an alternative to the CD137-specific antibodies to stimulate antitumor T cell responses. Shirwan lab reported that a streptavidin-conjugated murine CD137L (SA-mCD137L) complex could induce effective antitumor immune responses (67, 68). SA-mCD137L induces less pathological side effects than anti-CD137 agonistic antibody therapy, suggesting a higher therapeutic index of SA-mCD137L. Previously, we demonstrated that recombinant SA-hCD137L enhanced the cytotoxic effect of Vγ9Vδ2- T cells against influenza virus infection (37). Here, we further found that SA-hCD137L restored the antitumor activity of Vγ9Vδ2-T cells compromised by the IL-10-mediated TME. These data indicate that SA-hCD137L can provide an alternative to anti-CD137 agonistic for anti-tumor therapy.

There are no Vγ9Vδ2-T cells in mice, thus it is impossible to study these cells in mouse models (69). Previously, we successfully established humanized mice with a similar proportion of Vγ9Vδ2-T cells in murine peripheral blood to that in humans (12, 14, 70, 71). Importantly, here the synergistic effect of PAM and recombinant SA-hCD137L Vγ9Vδ2-T cells was verified in humanized mice.

In conclusion, our study further elucidates the role of CD137 in the antitumor activity of human Vγ9Vδ2-T cells in the IL-10-mediated immunosuppressive TME. The combination of a phosphoantigen and CD137 agonist also provides a novel strategy for treating EBV-induced tumors by avoiding Vγ9Vδ2-T cell exhaustion and enhancing the efficacy of Vγ9Vδ2-T cell-based therapy.

## Author Contributors

YP, WT, and YL conceived and designed the study, interpreted the results, wrote and edited the manuscript; YP, KW, ZX, CT, XW, YZ, and XM designed and performed the experiments, analyzed the results. WT supervised this study. All authors contributed to the article and approved the submitted version.

## Data Availability Statement

The original contributions presented in the study are included in the article/supplementary material. Further inquiries can be directed to the corresponding author.

## Ethics Statement

The studies involving human participants were reviewed and approved by the institutional review board of The University of Hong Kong/Hospital Authority Hong Kong West Cluster. The patients/participants provided their written informed consent to participate in this study. The animal study was reviewed and approved by the Committee on the Use of Live Animals in Teaching and Research, The University of Hong Kong.

## Funding

This study was supported partially by GRF, RGC (17122519, 17126317); Health and Medical Research Fund, Food and Health Bureau, Hong Kong SAR Government (18192021); Seed Funding for Strategic Interdisciplinary Research Scheme, the University of Hong Kong; Hong Kong SAR, China.

## Conflict of Interest

The authors declare that the research was conducted in the absence of any commercial or financial relationships that could be construed as a potential conflict of interest.

## Publisher’s Note

All claims expressed in this article are solely those of the authors and do not necessarily represent those of their affiliated organizations, or those of the publisher, the editors and the reviewers. Any product that may be evaluated in this article, or claim that may be made by its manufacturer, is not guaranteed or endorsed by the publisher.
